# Olfactory and gustatory chemical sensor systems in the African turquoise killifish: Insights from morphology

**DOI:** 10.1007/s00441-024-03923-5

**Published:** 2024-10-21

**Authors:** Daniela Giaquinto, Elisa Fonsatti, Martina Bortoletti, Giuseppe Radaelli, Elena De Felice, Paolo de Girolamo, Daniela Bertotto, Livia D’Angelo

**Affiliations:** 1 Department of Veterinary Medicine and Animal Production, University of Naples Federico II, Via F. Delpino, 1 I-80137 Naples, Italy; 2 Department of Comparative Biomedicine and Food Science (BCA), University of Padova, Viale Dell’Università 16, Legnaro, 35020 Padua, Italy; 3 School of Biosciences and Veterinary Medicine, University of Camerino, 62032 Camerino, Italy

**Keywords:** Taste buds, Olfactory epithelium, Fish

## Abstract

Smell and taste are extensively studied in fish species as essential for finding food and selecting mates while avoiding toxic substances and predators. Depending on the evolutionary position and adaptation, a discrete variation in the morphology of these sense organs has been reported in numerous teleost species. Here, for the first time, we approach the phenotypic characterization of the olfactory epithelium and taste buds in the African turquoise killifish (*Nothobranchius furzeri*), a model organism known for its short lifespan and use in ageing research. Our observations indicate that the olfactory epithelium of *N. furzeri* is organized as a simple patch, lacking the complex folding into a rosette, with an average size of approximately 600 µm in length, 300 µm in width, and 70 µm in thickness. Three main cytotypes, including olfactory receptor neurons (CalbindinD28K), supporting cells (β-tubulin IV), and basal cells (Ki67), were identified across the epithelium. Further, we determined the taste buds’ distribution and quantification between anterior (skin, lips, oral cavity) and posterior (gills, pharynx, oesophagus) systems. We identified the key cytotypes by using immunohistochemical markers, i.e. CalbindinD28K, doublecortin, and neuropeptide Y (NPY) for gustatory receptor cells, glial fibrillary acidic protein (GFAP) for supporting cells, and Ki67, a marker of cellular proliferation for basal cells. Altogether, these results indicate that *N. furzeri* is a microsmatic species with unique taste and olfactory features and possesses a well-developed posterior taste system compared to the anterior. This study provides fundamental insights into the chemosensory biology of *N. furzeri*, facilitating future investigations into nutrient-sensing mechanisms and their roles in development, survival, and ageing.

**Supplementary Information:**

The online version contains supplementary material available at 10.1007/s00441-024-03923-5.

## Introduction

Chemosensation is an ancient sense enabling animals to locate nutritious food and suitable mating partners and to avoid being eaten by predators or eating toxic substances. Chemosensory systems such as smell and taste are distinguished from the other senses by the qualitative heterogeneity of the stimuli: the chemical senses are responsible for detecting molecules of immense chemical variety (Mombaerts 2004). The recognition of chemical substances requires a repertoire of receptors, to match the diversity in chemical structures (Mombaerts 2004). A discrete high degree of evolutionary conservation of smell and taste receptors system is reported across vertebrates (Korsching 2020), despite the different evolutive pressures, ecological adaptations, and lifestyle habits of each species. For instance, homologous taste receptors, either type 1 and type 2 (T1Rs and T2Rs, respectively), of mammals have been identified in several fish genomes (Ishimaru et al. 2005) and are activated in response to certain chemical cues in a comparable way. As proof, mammalian T1R1/T1R3 heteromer detects umami substances (amino acids) likewise fish T1R1/T1R3 heteromer does in response to l-amino acids, such as l-Arg and l-Ser in a dose-dependent manner (Okada 2015).

From the anatomical standpoint, in fish species, the sense of smell is restricted to the olfactory rosette which is lined with the olfactory neuroepithelium projecting toward the olfactory bulb, the first brain relay of olfactory stimuli. In macrosmatic fish species, the sensory surface is folded to increase its surface and presents as several to many dozens of lamellae, emerging from a central midline or median raphe (Korsching 2020). Zebrafish, for instance, possess such a typical bilaterally symmetrical rosette, with 6–7 lamellae on each side in young adults and up to 10–11 lamellae per side in old adults (Hansen and Zeiske 1998; Villamayor et al. 2021). In microsmatic species, the olfactory sensory surface may be a simple patch (Yamamoto 1982; Yasuoka et al. 1999).

On the other hand, the sense of taste is transduced by cell receptors organized in onion-shape structures, called taste buds (TBs). On a morphological basis, in fish, three morphological types have been distinguished (Kasumyan 2019): type I protrudes above the surrounding epithelium and have a depression around its base, type II lacks the depression, and type III occurs within a pore on the flat epithelium (Reutter et al. 1974). TBs in fish display a wider distribution, including the skinhead, lips, oral, and pharyngobranchial regions. Type I and type II TBs are located mostly between the conical teeth on dentary, maxilla, vomer, tongue, and gills (Hara et al. 1994). Such distribution increases the probability that chemical contents which come out from punctured prey will not be diluted, but easily reach and contact taste receptors. Type III TBs are usually located in oropharyngeal zones associated with molar form teeth that fishes use to press, crush, and grind food during mastication (Lauder and Liem 1983; Devitsina 2005; Elsheikh et al. 2012).

Furthermore, based on the regional distribution, many authors outlined an anatomical segregation between an anterior and a posterior taste system, based on the taste bud distribution and innervation (Atema 1971; Finger and Morita 1985; Ikenaga et al. 2009). Accordingly, the distinction between anterior and posterior taste systems is as follows: TBs in the skin, lips, and anterior part of the mouth belong to the anterior taste system and are innervated by the facial nerve (VII) and functionally involved in food localisation, whereas TBs in the posterior part of the mouth and the gill arches belong to the posterior taste system and are innervated by the glossopharyngeal and vagal nerves (IX and X). These taste cells and nerves are involved in determination of palatability, i.e. whether to swallow or reject food items.

*Nothobranchius furzeri*, also known as African turquoise killifish, is now worldwide used model organisms in different research settings. Primarily employed as a model in ageing research, thanks to its short lifespan (maximum one year depending on the strain) which recapitulates typical phenotypic hallmarks of ageing (D’Angelo et al. 2016), it is gaining popularity also in ecotoxicological and developmental studies (Riddle and Hu 2021; Thoré et al. 2021). Much is known about the age-related aspects of morphology of *N. furzeri*, in relation to different systems, organs, and tissues (Cellerino et al. 2016) and about key aspects of its home-based behavioural repertoire (Mariën et al. 2024). Given the feeding habits, the African turquoise killifish should be considered a very selective feeder or picky eater. This fish species, indeed, does not eat all food items it comes across and shows a low degree of acceptance when exposed to a diet different from that normally administered. These observations are further corroborated by the challenges to define the most suitable diet in terms of nutrient composition and palatability (Žák et al. 2020, 2022).

A deep morphological analysis of the anatomical organization of chemical senses in the African turquoise killifish is missing. We therefore undertake this study to characterize the architecture and organization of chemical senses in adult specimens of *N. furzeri*, in the attempt to gain fundamental knowledge on the biology of the species and help future research in the comprehension of the related nutrient-sensing mechanisms which are key in the development, survival, and ageing process of living organisms. The novelty of our findings shed light on the following:

i) Anatomical characterization of olfactory epithelium and the cell type identification
ii) Organization of the taste system with its subdivision into an anterior and posterior system along with the cell type identification

Altogether, these findings are fundamental to develop future research on the sensory degeneration with huge consequences on the metabolic intake of living organisms.

## Materials and methods

### Animals and tissue preparation

All experiments were performed on *Nothobranchius furzeri* belonging to the long-lived strain MZM 04/10 at 16–20 weeks post hatching (wph), corresponding to an adult age. Authorization for tissue sampling was carried out in accordance with the Italian legislative Decree (n 26/2014) (N° 291/2022-PR). Fish were euthanized with an overdose of anesthetic, around 10 a.m. in a fasted state, to avoid the effects of circadian rhythms and feeding. Fish were placed for approximately 5–10 min in a methanesulfonate solution (MS-222, Ethyl 3-aminobenzoate methanesulfonate, cat# E10521, Sigma-Aldrich, Saint Louis, USA) at a concentration of 300 mg/L, until no vital signs (body and operculum movement, righting reflex) were observed.

Histological analyses were conducted on three male specimens of *N. furzeri*. The tissues were fixed in Bouin solution for 48 h. For paraffin embedding, tissues were dehydrated in a graded ethanol series, cleared with xylene, and embedded in paraffin. Serial transversal 7-µm-thick sections were cut at the microtome (Leica RM 2125, Nussloch, Germany).

### Histological staining and immunohistochemistry

Serial sections were stained with (i) haematoxylin/eosin (Histo-Line Laboratories HAE-2) for morphological count of TBs and (ii) Cresyl Violet (Sigma-Aldrich cat# C5042-10G) and Alcian Blue (pH 1 and pH 2.5) (Sigma-Aldrich cat# A3157-25G) for the description of the olfactory epithelium and TBs. The scanning system Scanscope 3DHISTECH Pannoramic SCAN II (Erpredia, Budapest, Hungary) was used to acquire the sections. For immunohistochemistry, the paraffin slides were deparaffinized in xylene and rehydrated in progressively diluted alcohols. Then, the slides were treated for 30 min with 3% H_2_O_2_ and, after washing with 1 × phosphate-buffered saline (PBS), were incubated in normal goat serum (Abcam cat# ab138478, 1:5 in 1 × PBS), at room temperature (RT) for 30-min incubation followed with a primary antibody (Table 1), at 4 °C overnight (on). Sections were then rinsed in 1 × PBS for 15 min and then incubated with Ultrapolymer Goat anti-rabbit/mouse IgG (H&L) conjugated to HRP (ImmunoReagents cat# UNIHRP-015), for 1 h at RT. Immunoreactive sites were visualized using a fresh solution of 10 mg of 3,30-diaminobenzidine tetrahydrochloride (DAB, Sigma-Aldrich, cat#D5905) in 15 mL of a 0.5 M Tris buffer.

**Table 1:** Used antibodies for immunohistochemistry

Antibody	Clonality	Dilution	Antigen retrieval	Manufacturer	Target cell
Calbindin D28k	Polyclonal	1:1000	Heat citrate pH 6 microwave	Invitrogen Pa1-931	OSN—TRC
Beta-tubulin	Polyclonal	1:150	Heat citrate pH 6 microwave	Cell Signaling 2146	Supporting cell—nerve
Ki-67	Polyclonal	1:200	Heat citrate pH 6 microwave	Abcam 15580	Basal cells
Npy	Polyclonal	1:1000		Abcam 30914	TRC
DCX	Polyclonal	1:100		Abcam 18723	TRC
Gfap	Monoclonal	1:100		Sigma-Aldrich G3893	Supporting cell

For double immunofluorescence, after dewaxing, the sections were rinsed in 0.1 M PBS every 5 min for three times and pre-incubated for 1 h at RT with the blocking solution (1:5 goat serum and PBS) and then incubated with the first primary antibody Calbindin D28K (1:100, cat# PA1-931, Invitrogen) for on at 4 °C in a humid chamber. Then, the sections were washed in PBS every 5 min for three times and incubated with Rhodamine Red-X AffiniPure Fab Fragment Goat Anti-Rabbit IgG (H + L) conjugated to tetramethylrhodamine-5-(and 6) isothiocyanate fluorochrome (1:200, cat# 111–297-003, Jackson ImmunoResearch, Cambridge, UK) for 2 h at 37 °C. Thereafter, the sections were rinsed in PBS and incubated with β-tubulin (1:150, cat#2146, Cell Signaling) at 4 °C in a humid chamber. After rinsing in PBS, the sections were treated with Fluorescein (FITC) AffiniPure Goat Anti-Rabbit IgG, F(ab′)₂ fragment specific conjugated to fluorescein isothiocyanate fluorochrome (1:50, cat# 111–095-006, Jackson ImmunoResearch, Cambridge, UK) for 2 h at 37 °C. Finally, the sections were washed with PBS and mounted with fluoroshield mounting medium with DAPI (cat #ab104139 Abcam). The images were acquired with a microscope. Images were observed and analysed with Leica—DM6B (Leica,Wetzlar, Germany) and processed with LasX software (Leica, Wetzlar, Germany). Digital raw images were optimized for image resolution, contrast, evenness of illumination, and background using Adobe Photoshop CC 2018 (Adobe Systems, San Jose, CA, USA).

### Specificity controls

For immunohistochemistry, both negative and positive controls were performed. Negative controls were achieved by substituting each primary antibody with normal serum during the specific step. For positive controls, we used the following samples: goat kid duodenum for calbindin and NPY (De Felice et al. 2021), the brain of *N. furzeri* for NPY (Giaquinto et al. 2022), the rat brain for Ki67 and Calbindin, zebrafish gut for tubulin, oral cavity for GFAP (Supplementary Information), and the African turquoise killifish brain for DCX (Terzibasi et al. 2012; Leggieri et al. 2022).

For immunofluorescence, negative controls were performed by omitting the primary antibody during the specific step (Supplementary Information).

### Quantifications of olfactory epithelium volume and TBs’ number

We conducted the morphological analysis of the olfactory epithelium using the methodology outlined by Hu et al. (2019). To determine the dimensions of the olfactory epithelium, we calculated the length by multiplying the number of sections containing the olfactory epithelium by the section thickness (7 µm). For the width and thickness of both the left and right olfactory epithelium, measurements were taken at 25%, 50%, and 75% along the rostro-caudal length of the epithelium. Regarding width, measurements were made at the midpoint between the apical part of the epithelium and the basement membrane. Thickness was measured at the midpoint of the olfactory epithelium, spanning from apical cells to the basement membrane. The means of these measurements were then considered for analysis. TB quantification was conducted on the skin, mouth, pharynx, gill arches, and oesophagus. To accurately estimate the number of TBs per organ, finely anatomical observations provided the inclusion and/or exclusion criterion. We indeed considered the transition between the region of mouth/pharynx and the gill arches by noting respectively the absence or presence of the gills and the transition between the region of pharynx/gill arches and the oesophagus by observing changes in the epithelium. Specifically, in the case of oesophagus, the presence of the tunica muscularis was a clear anatomical remark.

To determine the diameter of each taste bud, we identified 10 TBs for each of the target organs on serial sections. The diameter size was then calculated by multiplying the number of sections containing the taste bud by 7 µm (section thickness). Upon acquiring this data, we conducted TB counts every four sections to ensure comprehensive and non-redundant enumeration. Subsequently, we calculated the total number of TBs for each target organ.

## Results

### Morphological characterization of olfactory epithelium of the turquoise killifish

**Fig. 1:**
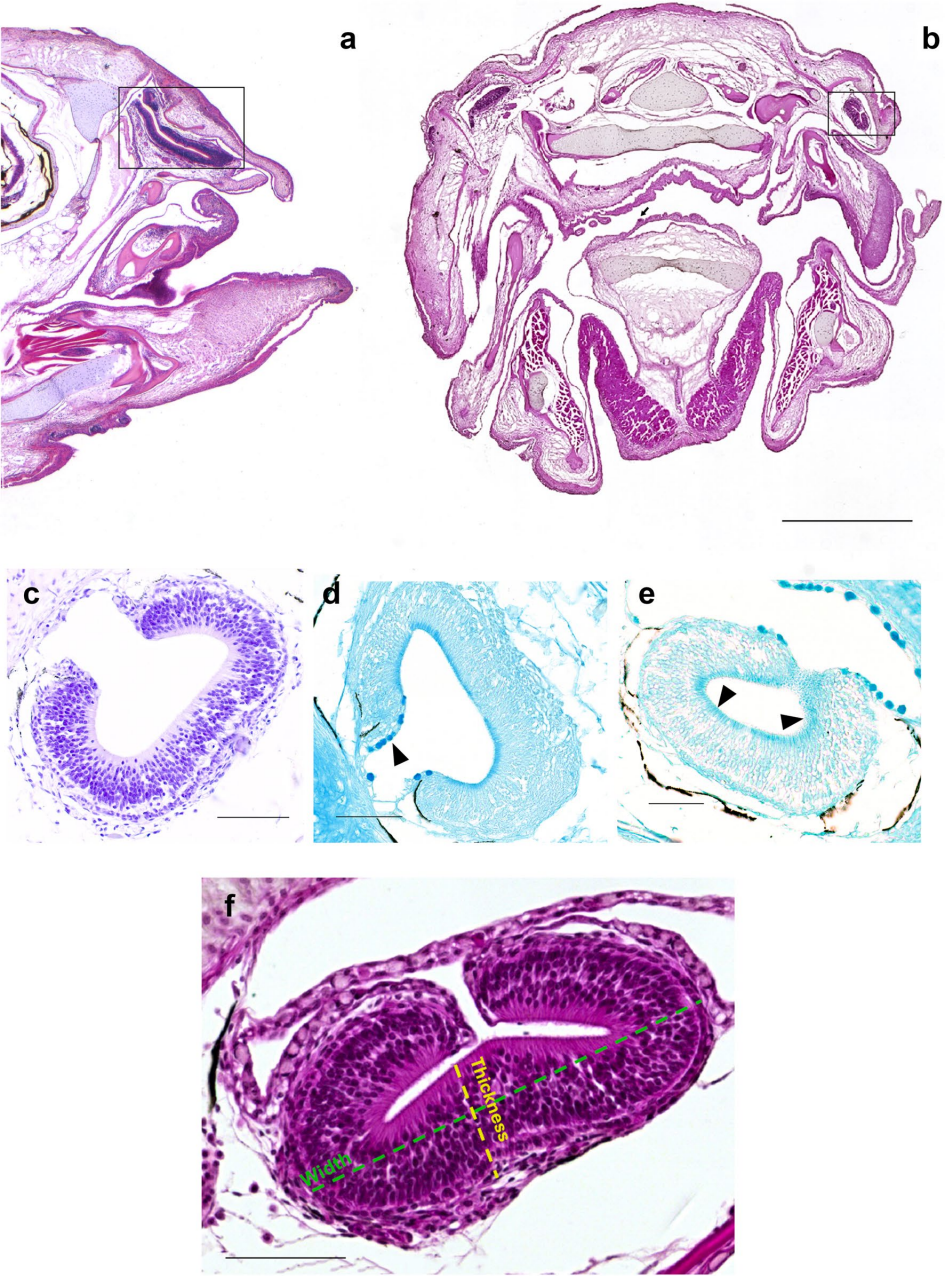
Overview of adult *N. furzeri* heads. **a** Haematoxylin–eosin staining of sagittal section of the whole head showing the olfactory epithelium (rectangle). **b** Haematoxylin–eosin staining of transversal section showing the olfactory epithelium (rectangle) and a taste bud localized along the oral cavity epithelium (arrow). **c** A magnified view of Cresyl Violet staining of transversal section of the olfactory epithelium (rectangle in **b**) to highlight sensory cells. **d** A magnified view of transversal section (rectangle in **b**) displaying goblet cells and acid mucins (arrowhead). **e** A magnified view of transversal section (rectangle in **b**) displaying sulphated mucins (arrowheads). **f** A magnified view of transversal section of the olfactory epithelium displaying two of the measurements (thickness and width) used to estimate the olfactory epithelium dimension. Scale bar: **a**, **b** 1 mm, **c**–**e** 200 µm, **f** 500 µm

The morphological staining has revealed that at the rostral edge of the olfactory chamber (Fig. 1a, b), the olfactory epithelium is relatively flat (Fig. 1b–f) with a slight elevation in the centre (Fig. 1f). The olfactory epithelium is close to the anterior nostril and also lines the lateral and dorsal sides of the nasal cavity. The olfactory epithelium is absent from the roof of the olfactory chamber but present along the ventral, lateral, and medio-dorsal surfaces. The staining confirms a distinct and simple organizational pattern characterized by a patch, lacking the folded sensory surface with lamellae. Sensory cells were highlighted using Cresyl Violet staining (Fig. 1c). The presence of goblet cells was investigated using Alcian Blue staining, at pH 2.5 for acid mucins (Fig. 1d) and at pH 1 for sulphated mucins (Fig. 1e). Olfactory epithelium measurements have been conducted as described above in the “Materials and methods” section. Details on measurements are described in Fig. 1f. On average, analysed olfactory epithelia from the three animals subjected to the analysis exhibited a length of 540.17 µm, a width of 325.12 µm, and a thickness of 71.14 µm, considering both the left and the right side of the head.

**Table:** 

Thickness (µm)	Width (µm)	Length (µm)
71.14	325.12	540.17

### Cellular characterization of the olfactory epithelium

**Fig. 2:**
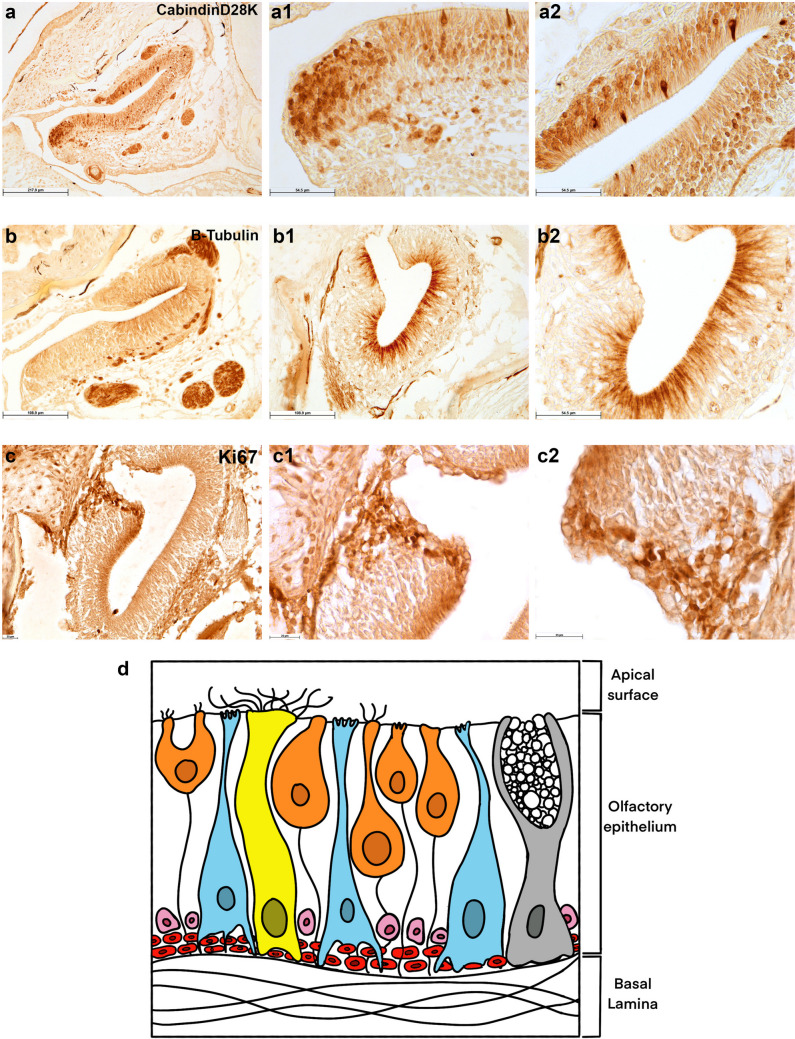
CalbindinD28K, β-tubulin, and Ki67 in the olfactory epithelium. **a** Overview of the olfactory epithelium, with CalbindingD28K distributed in globose-like cells in the posterior region and in some sparse microvillous sensory neurons. **a1** Higher magnification of CalbindingD28K immunoreactivity in globose-like cells in the posterior region and in some sparse microvillous sensory neurons. **a2** Higher magnification of CalbindingD28K immunoreactivity in some sparse microvillous sensory neurons. **b** Overview of the olfactory epithelium, with β-tubulin distributed in supporting cells and in neuronal fibres. **b1**, **b2** Higher magnification of β-tubulin distributed in supporting cells. **c** Overview of the olfactory epithelium, with Ki67 distributed in the cytoplasm of basal cells in the posterior region and in close proximity of the basal lamina. **c1**, **c2** Higher magnification of Ki67 distributed in the cytoplasm of basal cells in the posterior region. **d** Schematic drawing of the cytotypes identified in the olfactory epithelium: orange, sensory neurons; light blue, sustentacular cells; red, globose basal cells; pink, basal cells; yellow, ciliated cells; and grey, goblet cells. Scale bar: **a** 217 µm, **a1**, **a2**, **b2** 54.5 µm, **b**, **b1** 108.9 µm, **c**–**c2** 25 µm

**Fig. 3:**
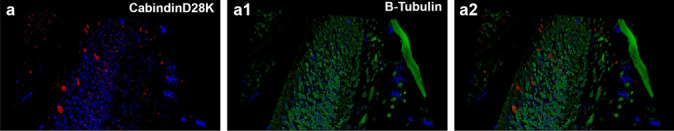
CalbindinD28K and β-tubulin are not co-localized in the olfactory epithelium. **a** Calbindin in sensory neurons. **a1** β-Tubulin in supporting cells. **a2** The two markers are not co-localized. Nuclei are counterstained by DAPI. Scale bar: 220 µm

The olfactory epithelium of teleost fish consists of three main types of cells: olfactory receptor neurons (ORNs), supporting or sustentacular cells, and basal cells, which are capable to divide and regenerate the epithelium after injury (Iqbal and Byrd-Jacobs 2010). We employed the marker CalbindinD28K (Bastianelli and Pochet 1994) to identify ORNs. In the African turquoise killifish, immunoreactivity to Calbindin was seen in ovoid-shaped olfactory neurons (Fig. 2a, a1, a2), mainly in the posterior part of the olfactory epithelium, and in neurons presenting a discretely elongated soma and short dendrites distributed along the superficial layer (Fig. 2a, a1, a2). Remarkably, the antibody also marked neuronal fibres in the proximity of the olfactory epithelium (Fig. 2a). β-tubulin IV, ubiquitous in the cytoskeleton of all cells (Holbrook et al. 2011), was used as a marker of supporting cells. β-tubulin immunoreactive cells showed a stretched shape from the epithelial surface to the basal lamina, forming a palisade monolayer and embedding the olfactory receptor cells (Fig. 2b, b1, b2). The apical surface seems to be covered with microvillous-like protrusions. Remarkably, the same antibody stained also neuronal fibres in the proximity of the olfactory epithelium (Fig. 2b). To confirm the cell-specific marker, we performed immunofluorescence staining and observed a clear specific signal against the two antibodies (Fig. 3a, a1, a2). Finally, Ki67 was used as a marker to identify the basal cells, which are dividing cells (Lemons et al. 2020), and were present in some clusters in the posterior area of the olfactory pitch (Fig. 2c, c1, c2). A schematic drawing of the different olfactory epithelium cytotypes, as identified by the specific markers, is reported in Fig. 2d.

### Anatomical distribution of taste buds in the turquoise killifish

**Fig. 4:**
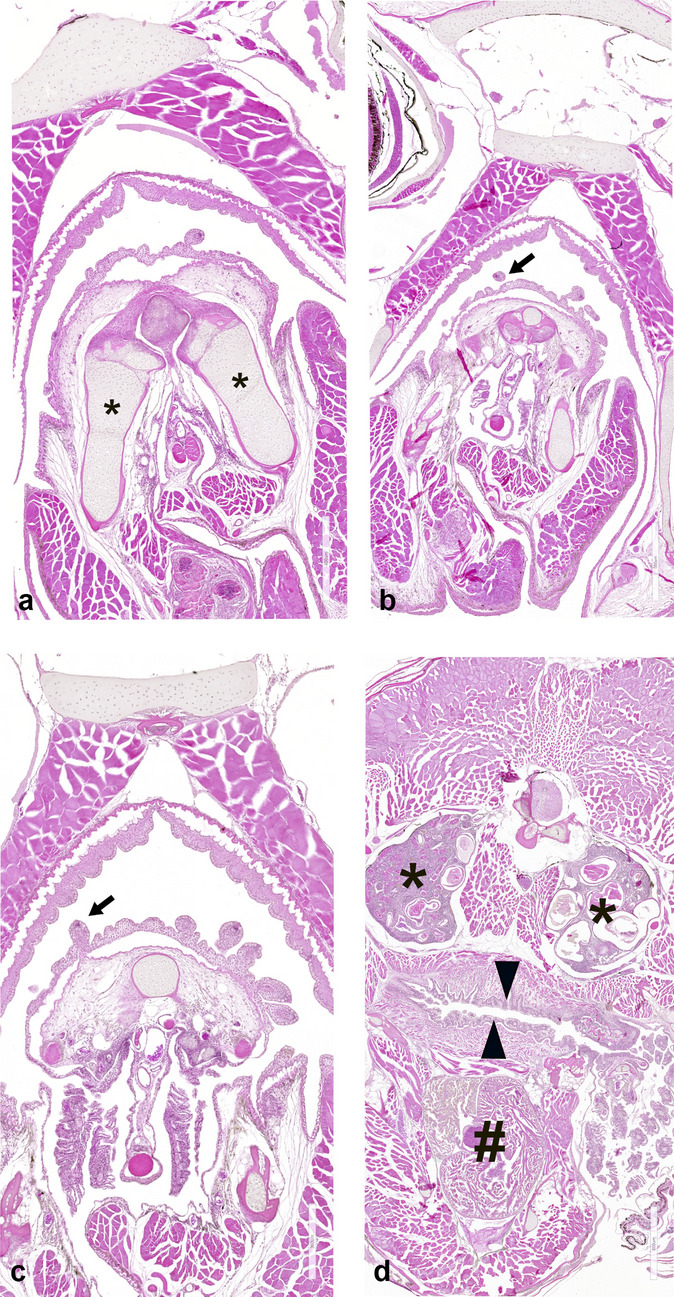
Transversal sections of the whole head of *N. furzeri* to identify the distribution of taste buds. **a** The most rostral part of the lips and oral cavity, as delineated also by the mandible (asterisks). **b** The most caudal part of the oral cavity, with the palate epithelium lined by TBs (arrow). **c** The pharyngeal cavity with the epithelium lined by TBs (arrow). **d** The region between the pharynx/gill arches and the oesophagus epithelium (triangles). The cephalic kidney (asterisks) and heart (gate) are clearly visible. Scale bar: **a** 400 µm, **b** 500 µm, **c** 300 µm, **d** 800 µm

**Fig. 5:**
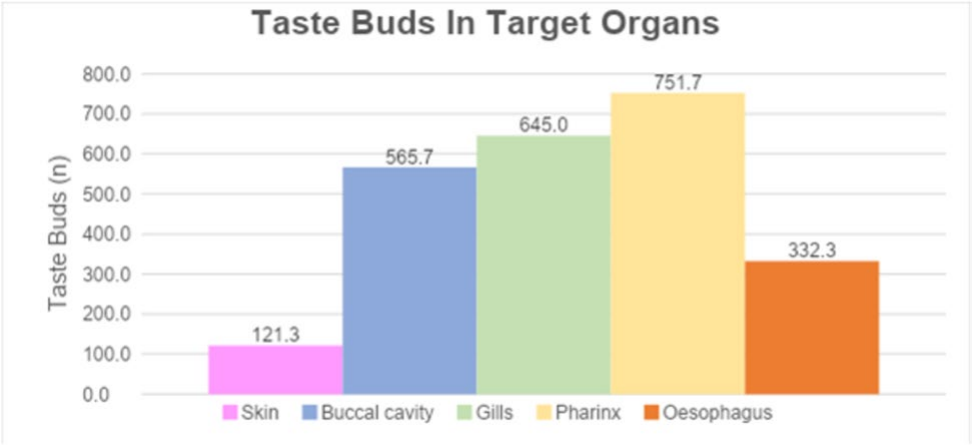
Average distribution of taste buds in the target organs

**Fig. 6:**
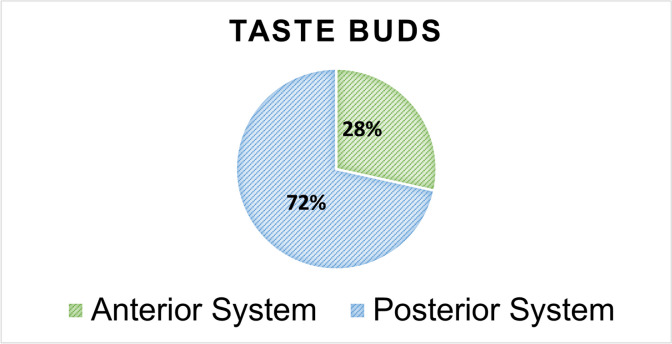
Quantification of taste buds in the head, subdivided into anterior and posterior systems

**Fig. 7:**
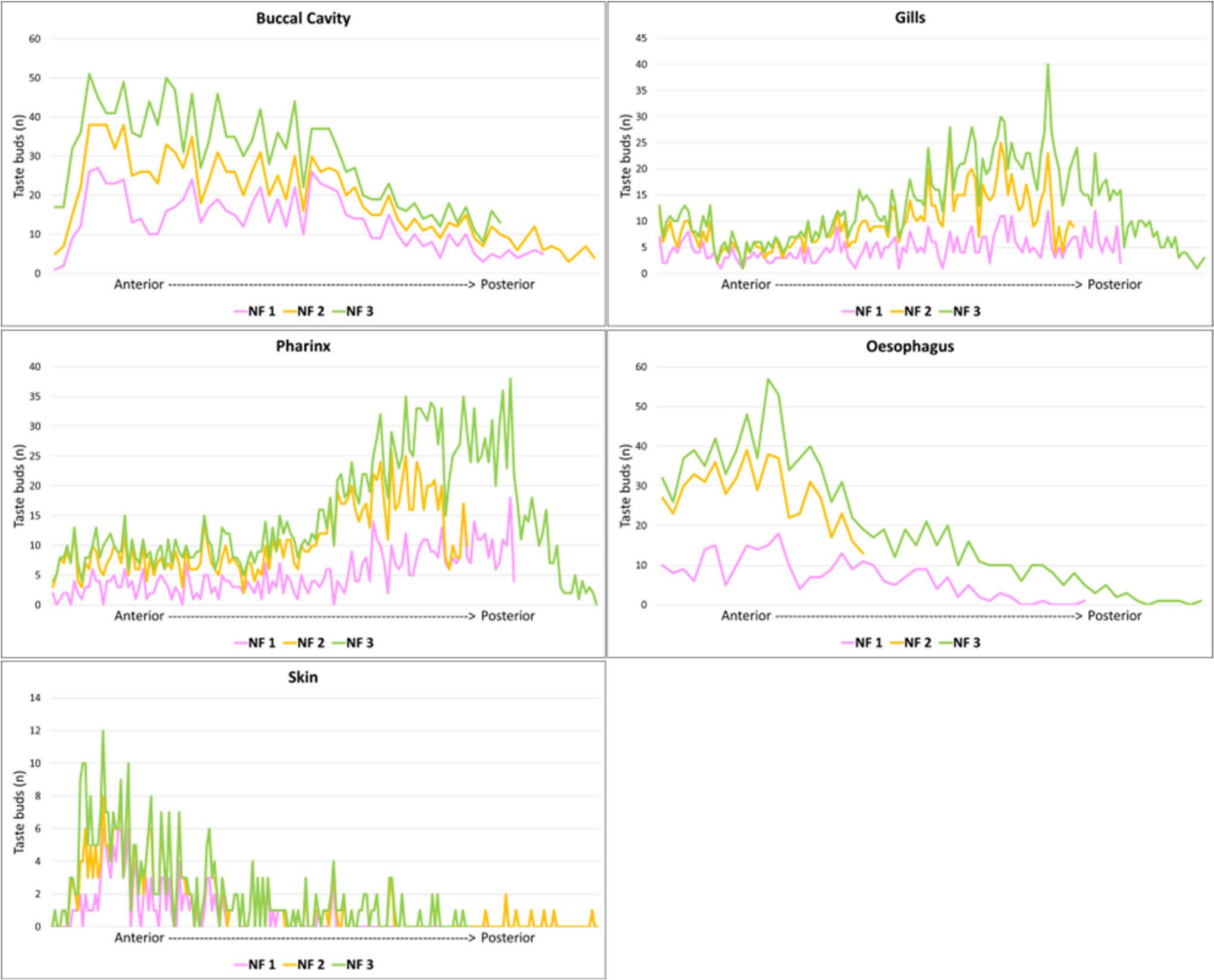
Overall distribution of TBs across the target organs, from anterior to posterior region. A common trend is observed for the three animals in each target organ. In the buccal cavity, the anterior part (from the lips to the caudal part of the mouth) shows a decreasing trend. Conversely, in the gills and pharynx, the trend is increasing toward the caudal part. In the oesophagus, the number of TBs decreases from anterior to posterior. In the skin, it is evident that the lips (anterior part) have the highest concentration of TBs. Pink, yellow, and green lines identify the three individuals used for TB quantification and distribution

TBs (Fig. 1b) appeared distributed in the skin covering the whole head and along the epithelium lining the mouth and lips, and more caudally along the epithelia of the gills, pharynx, and oesophagus. In our model species, we have quantified the number of TBs and distinguished in an anterior and posterior system. Each area was identified based on the morphological criteria. As previously described, morphological criteria were considered to identify the different organs. In Fig. 4, the appearance of the gills can be observed between the mouth and the region of the pharynx/gill arches (Fig. 4a–c). In Fig. 4, the transition between the region of the pharynx/gill arches and the oesophagus is visible (Fig. 4d). The counting on the three specimens confirms on average that the anterior system is composed of 687 TBs, of which 121 localized in the skin of the head and 565 in the oral cavity, whereas the posterior system comprehends on average 1729, of which 645 were in the epithelium of gills, 751 along the pharyngeal epithelium, and 315 in the oesophageal epithelium (Figs. 5 and 6). The count of TBs provided data on their distribution within each target organ, representing the presence of TBs from the anterior to the posterior region (Fig. 7).

### Cellular characterization of the taste buds

**Fig. 8:**
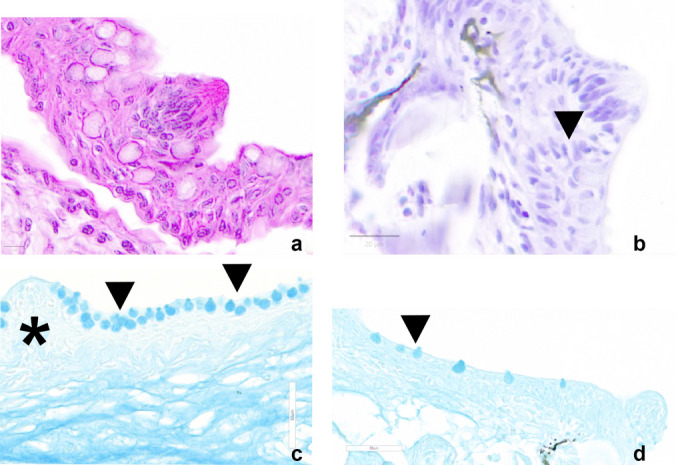
Transversal sections of taste buds. **a** Haematoxylin and eosin staining of basal cells of the taste bud and elongated receptor cells with the apical surface protruding toward the epithelial surface. **b** Cresyl Violet staining of receptor cells (arrowhead). **c** Alcian Blue staining, pH 2.5, of acid mucins (arrowheads) along the oral cavity epithelium around the taste bud (negative) (asterisk). **d** Alcian Blue staining, pH 1, of sulphated mucins less abundantly distributed along the epithelium (arrowhead). Scale bars: **a** 10 µm, **b** 20 µm, **c**, **d** 50 µm

The morphology of TBs was appreciated by histological staining via haematoxylin and eosin (Fig. 8a), Cresyl Violet (Fig. 8b), Alcian Blue pH2.5 (Fig. 8c), and pH1 (Fig. 8d). In Fig. 8a, basal cells identified by round cytoplasm at the base of the taste bud are identifiable along with elongated receptor cells with the apical surface protruding toward the epithelial surface. Only receptor cells are stained by Cresyl Violet (Fig. 8b). Alcian Blue staining, at pH 2.5, highlights the great amount of acid mucins (Fig. 8c) lining only the epithelium around the taste bud, whereas at pH 1, sulphated mucins are less abundant along the epithelium (Fig. 8d).

The TBs of teleost fish are made of three types of cells: gustatory or taste receptor cells (TRCs), supporting cells, and basal cells.

**Fig. 9:**
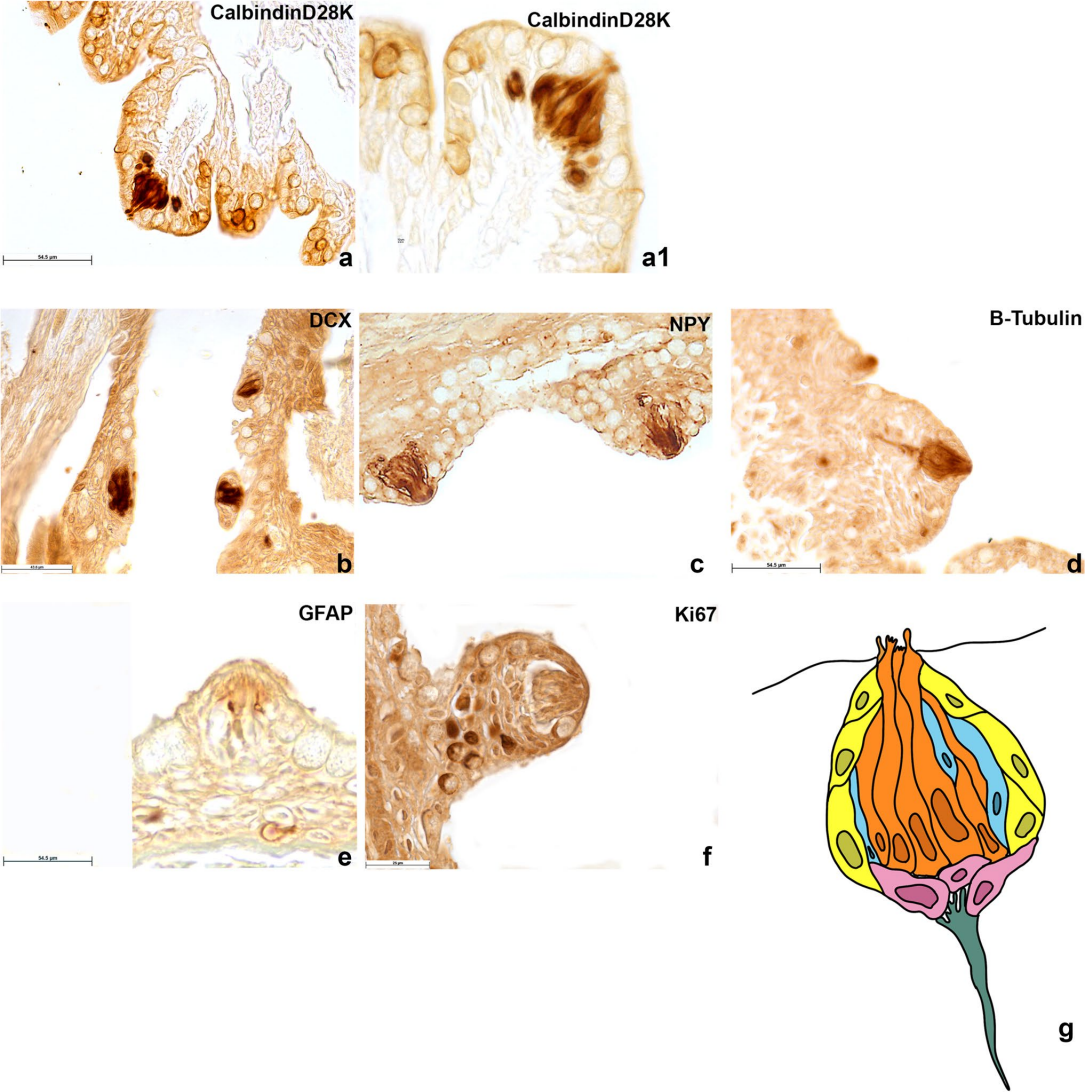
Calbindin28dK, DCX, NPY, GFAP, and Ki67 in the transversal sections of taste buds. **a**, **a1** Immunostaining of Calbindin28dK. **b**, **c** DCX and NPY in receptor cells. **d** β-Tubulin-IV immunoreactivity in receptor cells and in the sensory nerve fibres. **e** Immunoreactivity to GFAP in supporting cells with elongated shape intermingled among receptor cells. **f** Immunoreactivity to Ki67 in basal cells of the TBs. **g** Schematic drawing of the cytotypes identified in the taste bud. Orange: receptor cells; light blue: supporting cells; pink: basal cells; yellow: edged receptor cells; green: nerve fibre of the taste bud. Scale bar: **a**, **c**–**e** 54.5 µm, **a1** 10 µm, **b** 43.5 µm, **f** 25 µm

Both TRCs and supporting cells have an elongated shape and run more or less parallel, following the long axis of the taste bud. To better identify the TRCs in *N. furzeri*, we used the markers Calbindin28dK (Fig. 9a, a1), DCX (Fig. 9b), and NPY (Fig. 9c). All TRCs immunoreactive cells displayed an elongated shape occupying the long axis of the TB and reaching the surface of the epithelium through a pore with variable diameter. β-Tubulin-IV immunoreactivity was observed in TRCs of the taste buds and in the sensory nerve fibres (Fig. 9d).

As for supporting cells, immunoreactivity to GFAP (Fig. 9e) was observed in a few cells with elongated shape intermingled among TRCs. Basal cells were characterized by means of Ki67, which stained the cytoplasm of cells at the basis of the TBs (Fig. 9f).

A schematic drawing of the different taste buds cytotypes, as identified by the specific markers, is reported in Fig. 9g.

## Discussion

Chemical senses play a vital function of an animal as they are deeply involved in food intake, mating, and predatory avoidance behaviours. A wider diversity of the morphological organization of olfactory and taste senses is reported in teleost species, which are the largest groups of vertebrates, adapted to the most diversified ecological habitats (Volff 2005). As a proof, catfish species, *Ictalurus natalis*, possess more than 175,000 TBs over the entire body surface (Northcutt 2005), and salmonids lack external TBs but have the highest densities (over 30/mm^2^) in the oral cavity (Hara et al. 1994). Here, we describe for the first time the morphological organization of olfactory epithelium and TBs in the African turquoise killifish *N. furzeri*. This is a small freshwater fish species, inhabiting the temporary ponds which are formed during the rainy season in the eastern part of Africa (Cellerino et al. 2016) and subjected to the environmental fluctuations of the unpredictable environment as well as to the turbidity of the water. These ecological aspects may have certainly contributed to shape the morpho-physiological adaptation of chemical senses.

We observed that the olfactory sensory surface of the turquoise killifish appears organized as a simple patch, similarly to the closest related species medaka (Yasuoka et al. 1999) and guppy (Bettini et al. 2023), all Cyprinodontiformes species, but differently from zebrafish, which is characterized by the olfactory rosette (Korsching 2020). Notably, the nostrils opening is very narrow in turquoise killifish, unlikely appreciable under the stereomicroscope, suggesting that the water amount flowing in the nasal cavity and contacting the olfactory epithelium is very limited. Altogether, our morphometric observations prompt us to consider this species as microsmatic, according also to the evolutionary position as neoteleost (Kirchmaier et al. 2015).

With regards to the identification of the olfactory epithelium cytotypes, we have here mainly focused on the characterization between the three cell types which are reported in fish olfactory epithelium: sensory cells, supporting cells, and basal cells (Korsching 2020). Our observations outline the occurrence of the three cytotypes, which display their specific morphological features. As for sensory cells, CalbindinD28K immunoreactivity was indeed detected either in round-shaped cells, resembling the globose or crypt cells which have been depicted in zebrafish, or in sensory neurons, resembling the microvillous ciliated neurons (Ahuja and Korsching 2014). CalbindinD28K is a calcium-binding protein during both normal tissue development and regeneration of tissue. In mammalian olfactory epithelium, it has been widely investigated, and it is robustly referred as a marker of sensory neurons (Fujiwara et al. 1997; Jia and Halpern 2003).

Our immunohistochemical analysis suggests that β-tubulin might be considered a reliable marker to identify the supporting cells, whose cell bodies are more or less cylindrical, often with a pronounced basal part resting on the basal lamina. Finally, we also identified basal cells by using Ki67. This cytotype forms the reservoir for the growth and regeneration of olfactory sensory neurons (Lemons et al. 2020). Future morphological investigations are deemed necessary for a more in-depth cytotype characterization of the olfactory epithelium in the turquoise killifish.

TBs appear as pear- or onion-shaped distributed in the extra-oral and oral regions, and based on the innervation, we could clearly segregate the distribution into an anterior and posterior system, respectively innervated by facial nerve and glossopharyngeal vagal nerves, similarly to the dichotomy reported in mammals (Korsching 2020). In this study, we have morphologically identified only type I TBs, protruding above the surrounding epithelium and having a depression around its base (Kasumyan 2019). The taste bud quantification clearly documented that the number of the TBs belonging to the posterior system is more than doubled in comparison to the anterior. The posterior system is innervated by vagal nerves, whose main neurons are located in the vagal lobe. Not surprisingly, vagal lobes are well developed in the medulla oblongata of *N. furzeri* (D’Angelo 2013) although it does not show the complex laminar cytoarchitecture described in goldfish (Ikenaga et al. 2009). TBs belonging to the posterior system are responsible for mediating palatability and driving the decision whether to swallow or reject the food items. Therefore, the abundance of TBs in the posterior system may support the behavioural trait of turquoise killifish as “selective feeder” and should be well-considered when challenging the introduction of new diets in the fish management under standard laboratory conditions. Recently, efforts to diet standardization have been reported on juveniles and young adults (Žák et al. 2020) and adult animals (Žák et al. 2022). However, the authors did not explore the impact of food palatability as well as the involvement of chemosensation in food preference and acceptance. The current hypothesis underlying the foraging behaviour is based on the sight which is used to locate the prey, both in turbid or clean waters (Žák and Suhajovà 2024).

The identification of cytotypes populating the TBs was mainly addressed to label receptors, supporting and basal cells. For receptor cells, we employed CalbindinD28K which clearly labelled the sensory cells and can be thus considered a reliable marker for sensory cells in the chemical senses. This is considered a specific marker also in mammals (Miyawaki et al. 1997). Differently from olfactory epithelium, β-tubulin in the TBs seems to specifically stain sensory cells along with nerve fibres, being a key component of the axonal microtubules (Di Fan et al. 2019). Most interestingly, we tested DCX, an essential factor in neurogenesis and capable of modulating and stabilizing microtubules (Ayanlaja et al. 2017). This marker has been proposed for the first time in the brain of *N. furzeri,* which possesses the gene encoding for the protein, differently from zebrafish (Terzibasi et al. 2012), and we here show that can be used also as a marker of the taste receptor cells.

Furthermore, we confirm that also in our species, NPY can be used as a marker of sensory cells, as reported in the earliest vertebrates such as lampreys (Barreiro-Iglesias et al. 2008) as well as in mammals (Herness 2012). GFAP, typical of glial cells, abundantly expressed in the aged brain of turquoise killifish due to the increased age-associate gliosis (Terzibasi et al. 2012) was appreciated in supporting cells of TBs of this species. To our knowledge, GFAP is considered a marker of TBs supporting cells in mammals (Mii et al. 2014), and we used this marker in a fish species. Finally, Ki67 marker was used to identify basal cells (Jang et al. 2014).

In conclusion, this study provides a preliminary but important bulk of knowledge on the olfactory and gustatory chemical sensor systems in the fish species *N. furzeri.* We have here demonstrated that this is a microsmatic species, coherently with the fact that it belongs to the *Cyprinodontiformes*, and have identified the main cytotypes of the olfactory epithelium; the TBs’ posterior system is more developed compared to the anterior and tested a series of useful markers to distinguish between sensory, supporting, and basal cells. Altogether, our findings open new research opportunities in the field of the comparative evolution of chemical senses as well as the influence of the chemical senses on the body metabolic regulation.

## Supplementary Information

Below is the link to the electronic supplementary material.

Supplementary file1 (DOCX 2333 KB)

## Data Availability

No datasets were generated or analysed during the current study.
