# Supplementary material for: Olfactory and gustatory chemical sensor systems in the African turquoise killifish: Insights from morphology
Source: Cell Tissue Res. 2024 Oct 21;398(3):239–52. doi: 10.1007/s00441-024-03923-5 (PMC11615025; doi:10.1007/s00441-024-03923-5)

**Supplementary Information**

Specificity Controls

**Figure SI1. Specificity Controls of antibodies used in light and fluorescence microscopy. A.** Duodenum of goat kid used as positive control for NPY, with positive staining in the neuroendocrine cells of duodenal villi and in the enteric nervous system. **B.** Rat brain used as positive control for Ki67. **C.** Anterior part of the intestine of zebrafish used as positive control for β-Tubulin. **D.** Zebrafish taste buds used as positive control for GFAP. **E.** Negative control of CalbindinD28K in the taste buds of the African turquoise killifish. **F.** Rat brain used as positive control for Calbindin for fluorescence staining. **G.** Immunofluorescence negative control of Calbindin in the taste buds of the African turquoise killifish. Scale bar: A 12.5 µm, B 25 µm, C,E 108.9 µm, D,F 54.5 µm, G 20 µm.


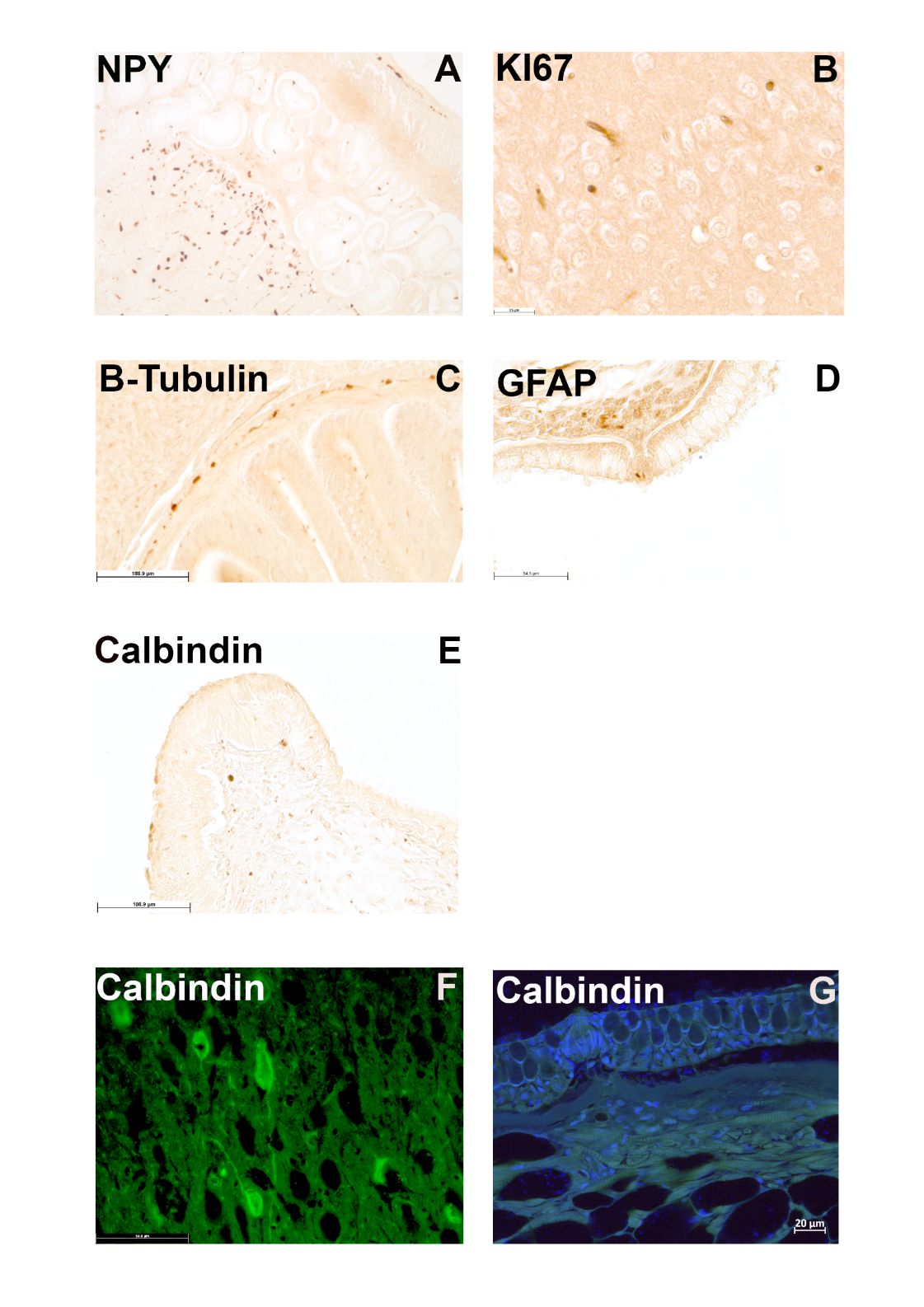

Supplement: Supplementary file 1 — Supplementary file1 (DOCX 2333 KB) [file 441_2024_3923_MOESM1_ESM.docx]
